# Contrast-Enhanced Ultrasound Combined with Methylene Blue for Sentinel Lymph Node Biopsy After Neoadjuvant Chemotherapy in Patients with Initially cN1 Breast Cancer: A Multicenter Prospective Cohort Study

**DOI:** 10.3390/cancers18152383

**Published:** 2026-07-24

**Authors:** Duanyang Zhai, Haiyun Tang, Yuanjian Fan, NiJiati AiErken, Mengmeng Zhang, Yawei Shi, Yunjian Zhang, Yanling Zheng, Zhen Shan, Ying Lin

**Affiliations:** 1 Department of Breast Surgery, The First Affiliated Hospital, Sun Yat-sen University, Guangzhou 510080, China; 2 Department of Breast and Thyroid Surgery, The Seventh Affiliated Hospital, Sun Yat-sen University, Shenzhen 518107, China; 3 Department of Ultrasound, The First Affiliated Hospital, Sun Yat-sen University, Guangzhou 510080, China

**Keywords:** breast cancer, sentinel lymph node biopsy, contrast-enhanced ultrasound, methylene blue, neoadjuvant chemotherapy

## Abstract

Background: Reliable axillary restaging after neoadjuvant chemotherapy (NAC) is essential for patients with initially cN1 breast cancer considered for surgical de-escalation. Although radioisotope plus blue dye dual-tracer mapping can reduce the false-negative rate (FNR) of sentinel lymph node biopsy (SLNB), its routine use is limited by radioisotope access, infrastructure requirements, and procedural complexity. We evaluated contrast-enhanced ultrasound (CEUS) combined with methylene blue as a non-radioactive dual-mapping strategy. Methods: In this two-center prospective cohort study, patients undergoing surgery after NAC were enrolled if they received preoperative CEUS, intraoperative methylene blue mapping, SLNB, and axillary lymph node dissection. The primary analysis focused on patients with initially cN1 disease, whereas cN2–3 patients were included only for exploratory analyses. Detection rate (DR), FNR, predictive values, CEUS–methylene blue concordance, and CEUS performance for predicting SLN metastasis were assessed. Results: A total of 269 patients were enrolled, including 208 with initial cN1 disease, 45 with cN2 disease, and 16 with cN3 disease. In the cN1 cohort, CEUS plus methylene blue achieved the highest SLN DR (99.04%; 95% CI, 96.56–99.74), followed by CEUS alone (98.08%) and methylene blue alone (71.63%). The FNR was 9.38% (95% CI, 4.37–18.98) for both CEUS plus methylene blue and CEUS alone, versus 12.50% for methylene blue alone. CEUS–methylene blue concordance was 91.16%. For predicting SLN metastasis, CEUS showed 65.52% sensitivity, 86.30% specificity, and 80.39% accuracy. No immediate procedure-related adverse events occurred. Conclusions: CEUS combined with methylene blue showed high SLN detection and a promising false-negative profile in patients with initially cN1 breast cancer after NAC. These findings support further evaluation of this feasible non-radioactive strategy with larger comparative studies, particularly in settings with limited access to radioisotope-guided mapping.

## Simple Summary

After preoperative chemotherapy, some patients with breast cancer may be able to avoid extensive axillary surgery if sentinel lymph nodes can be identified and assessed accurately. Standard mapping commonly relies on radioactive tracers, which are unavailable in many hospitals. We prospectively evaluated contrast-enhanced ultrasound combined with methylene blue as a non-radioactive mapping strategy after neoadjuvant chemotherapy. In patients with initially cN1 disease, the combined approach identified sentinel lymph nodes in 99.04% of cases and the false-negative rate was 9.38%. Contrast-enhanced ultrasound localization was highly concordant with methylene blue mapping and also provided supplementary information on nodal metastatic status. These findings suggest that this method may support further evaluation of a feasible non-radioactive axillary mapping strategy in selected patients.

## 1. Introduction

Breast cancer is the most commonly diagnosed cancer among women, and axillary nodal status remains central to disease staging, prognosis, and locoregional treatment planning [1]. In patients presenting with node-positive disease, neoadjuvant chemotherapy (NAC) can downstage both the breast and axilla, thereby creating opportunities to de-escalate breast and axillary surgery [2,3,4]. Axillary lymph node dissection (ALND) has long been the standard axillary surgical procedure. Because more than 40% of clinically node-positive patients may achieve an axillary pathological complete response (pCR) after NAC, whether ALND can be safely omitted has become an important clinical question. Recent studies have suggested that, in carefully selected patients with initially cN1 disease who convert to ycN0 after NAC, axillary recurrence remains very low after sentinel lymph node biopsy (SLNB) alone, and no significant differences in overall survival have been observed compared with ALND [5,6,7,8]. Accordingly, as systemic therapy continues to improve, the clinical focus has shifted from whether NAC is effective to how the axilla should be restaged after treatment and which patients may safely avoid ALND.

SLNB is the standard staging procedure for patients with clinically node-negative breast cancer [9,10]. It has also been extensively investigated after NAC in initially node-positive patients as a potential strategy for axillary de-escalation. However, the clinical acceptability of post-NAC SLNB depends on achieving both a sufficiently high detection rate (DR) and a sufficiently low false-negative rate (FNR). Landmark prospective studies have shown that the accuracy of post-NAC SLNB remains suboptimal when technical conditions are not adequately optimized. In ACOSOG Z1071, the FNR of SLN surgery after NAC in patients with biopsy-proven node-positive disease was 12.6%. Similarly, in the SENTINA trial, patients who converted from cN+ to ycN0 after NAC had a DR of 80.1% and an FNR of 14.2% [11,12]. Therefore, improving sentinel lymph node (SLN) detection while reducing false-negative results remains a key challenge for axillary de-escalation after NAC.

To reduce the FNR of SLNB in initially node-positive patients after NAC, clinical guidelines and prospective studies generally recommend strategies that include dual-tracer mapping with a radioisotope plus blue dye and retrieval of at least three SLNs [13]. Previous studies have shown that these optimization measures can significantly improve the accuracy of post-NAC SLNB and reduce the FNR to a level approaching or below the clinically acceptable range. Retrieval of the clipped node in ACOSOG Z1071 reduced the false-negative rate to 6.8%; the SN FNAC study reported a false-negative rate of 8.4% with mandatory immunohistochemistry; and the prospective SenTa study reported a false-negative rate of 4.2% for targeted axillary dissection [14,15,16,17,18,19,20]. However, these strategies often depend on radioisotopes, clip placement, additional imaging localization, or more complex perioperative workflows, thereby increasing technical complexity and imposing higher requirements on equipment, personnel training, and multidisciplinary coordination. As a result, their implementation in routine clinical practice, particularly in resource-limited settings, remains challenging. For many community hospitals and resource-constrained centers without access to radioisotope mapping, improving SLN detection while reducing false-negative results without relying on radioactive tracers remains a key issue in axillary de-escalation after NAC [21,22].

Against this background, there is a continuing need for a practical and reliable non-radioactive strategy for axillary staging after NAC. Methylene blue mapping has long been used as a practical intraoperative method for sentinel lymph node identification because of its simplicity, low cost, and broad availability, although its performance is suboptimal when used as a single tracer. Likewise, previous studies have demonstrated the feasibility of contrast-enhanced ultrasound (CEUS) for preoperative sentinel lymph node localization, showing that it can delineate lymphatic drainage pathways and identify the first enhanced node in real time without radioisotope guidance [23,24,25,26,27,28]. On this basis, combining methylene blue with CEUS may represent a rational strategy that integrates preoperative lymphatic mapping with intraoperative visual confirmation.

Accordingly, the present study aimed to evaluate the diagnostic performance and feasibility of CEUS combined with methylene blue for SLNB in patients with initially cN1 breast cancer after NAC, with a particular focus on its detection performance, false-negative rate, concordance between CEUS localization and methylene blue mapping, and the adjunctive role of CEUS in predicting SLN metastasis. Patients with initial cN2–3 disease were included only for exploratory analyses to assess the potential boundaries of this strategy in patients with higher initial nodal burden.

## 2. Materials and Methods

### 2.1. Study Design and Study Population

This was a two-center, prospective cohort study. The study was approved by the Ethics Committee of the First Affiliated Hospital of Sun Yat-sen University and the Seventh Affiliated Hospital of Sun Yat-sen University (approval number: [2021]318) and registered at ClinicalTrials.gov (NCT04932460) on 15 June 2021. All patients provided written informed consent.

**Figure 1:**
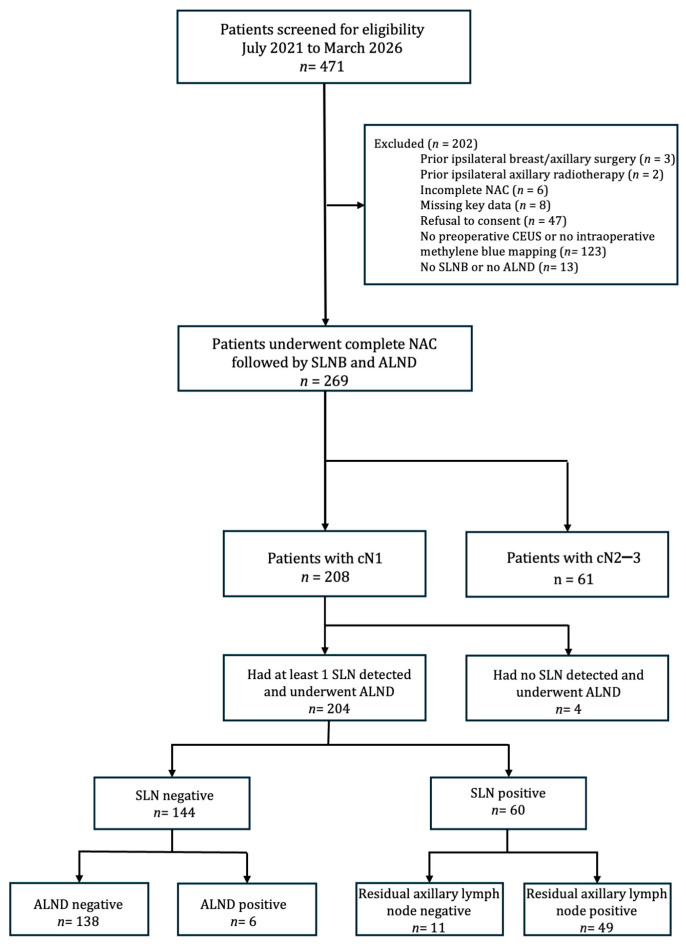
Flow diagram of the study. cN, clinical lymph node status before NAC; NAC, neoadjuvant chemotherapy; CEUS, contrast-enhanced ultrasound; ALND, axillary lymph node dissection; SLNB, sentinel lymph node biopsy; SLN, sentinel lymph node.

Consecutive breast cancer patients who underwent surgery after receiving standard NAC at the First Affiliated Hospital of Sun Yat-sen University and the Seventh Affiliated Hospital of Sun Yat-sen University between July 2021 and March 2026 were prospectively enrolled. The inclusion criteria were as follows: (i) female patients aged ≥18 years; (ii) histopathologically confirmed invasive breast cancer before NAC; (iii) initial clinical stage of cT0–4N1-3M0 before NAC; (iv) completion of a standard NAC regimen followed by breast and axillary surgery, including SLNB and ALND, at one of the participating centers; and (v) consent to undergo preoperative sentinel lymph node CEUS examination and intraoperative methylene blue mapping. Patients with other malignant diseases, prior ipsilateral breast or axillary surgery or radiotherapy, or missing key clinical or pathological data were excluded. Patients with initially cN1 disease constituted the primary analytic cohort because this group is the most clinically relevant population for post-NAC axillary de-escalation. Patients with initial cN2–3 disease were included only for exploratory analyses to evaluate the potential performance boundaries of this strategy in patients with higher initial nodal burden. The patient selection process is shown in Figure 1.

### 2.2. Study Endpoints

The primary endpoints of this study were the sentinel lymph node detection rate and the false-negative rate. DR was defined as the proportion of patients in whom at least one sentinel lymph node was successfully identified by CEUS, methylene blue, or the combined approach. FNR was defined as the proportion of patients with residual axillary nodal disease confirmed by ALND who had negative SLN findings. The secondary endpoints included: (i) the number of sentinel lymph nodes detected by each method; (ii) the sensitivity, positive predictive value (PPV), and negative predictive value (NPV) of each method; (iii) the DR and FNR in subgroups; (iv) the concordance between CEUS localization and methylene blue mapping; (v) the adjunctive diagnostic value of CEUS for lymph node metastasis; and (vi) procedure-related adverse events.

### 2.3. Sample Size Calculation

A precision-based approach was used for sample size estimation, and the larger estimate derived from the two endpoints, DR and FNR, was selected as the final required sample size. Based on previous post-neoadjuvant SLNB studies in initially node-positive breast cancer, the expected DR was set at 95%, with an allowable margin of error of 3% for the 95% confidence interval, and the expected FNR was set at 10.0% with an allowable margin of error of 5.0%. Assuming a residual axillary nodal positivity rate of approximately 40%, the minimum required sample size was estimated to be 348 patients. After accounting for an anticipated 10% rate of loss to follow-up or missing key data, the final target sample size was set at approximately 380–390 patients.

As updated clinical guidelines increasingly supported omission of ALND in selected patients with initially cN1 disease who converted to ycN0 after NAC and had negative SLNB findings, continued mandatory ALND raised ethical concerns. Following a joint review by the institutional ethics committees and the study steering committee, enrollment was terminated early to minimize surgical morbidity and avoid potentially unnecessary ALND. Consequently, the final sample size did not reach the originally estimated target, and the final cohort was underpowered relative to the original design, particularly for precise estimation of the primary FNR endpoint.

### 2.4. Neoadjuvant Chemotherapy Regimens

All patients received standard NAC regimens based on the National Comprehensive Cancer Network (NCCN) guidelines. The specific treatment regimen was determined according to tumor molecular subtype, clinical stage, and routine clinical practice at the time of treatment. Patients with HER2-negative disease primarily received anthracycline- and/or taxane-based chemotherapy, with carboplatin added in selected cases. Patients with HER2-positive disease received anti-HER2 targeted therapy in addition to chemotherapy. The number of NAC cycles and the specific drug regimen were adjusted according to individual patient characteristics and treatment response.

### 2.5. Study Procedures

All enrolled patients underwent preoperative sentinel lymph node CEUS within one week before surgery. All CEUS examinations were performed by two dedicated ultrasound specialists, each with more than 10 years of experience in breast imaging, and the imaging findings and final reports were jointly reviewed and confirmed by both operators. Three ultrasound systems were used: the Siemens ACUSON (Siemens Healthineers, Erlangen, Germany), Mindray Resona 7 (Mindray, Shenzhen, China), and Philips iU22 (Philips, Amsterdam, The Netherlands). Each system was equipped with a high-frequency linear-array transducer and contrast-specific imaging sequences. Examinations were performed in continuous real-time CEUS mode using a low mechanical index (MI < 0.15). The ultrasound contrast agent was SonoVue (Bracco, Milan, Italy), prepared by mixing the lyophilized powder with 5 mL of normal saline and shaking for 30 s before use. The contrast agent was injected subcutaneously into the outer upper quadrant of the areola on the affected side. After injection, the lymphatic drainage of the contrast agent from the periareolar area toward the axilla was observed in real time, and the location, number, and enhancement characteristics of the first or first few enhanced lymph nodes were recorded. On CEUS, SLNs were defined as the first one or several axillary nodes to enhance along the lymphatic drainage channels originating from the periareolar region. The following findings were recorded during the examination: (i) whether lymphatic drainage pathways were successfully visualized; (ii) whether sentinel lymph nodes were successfully identified; (iii) the number of sentinel lymph nodes detected by CEUS; and (iv) the enhancement patterns and suspicious metastatic features of the sentinel lymph nodes. The lymphatic drainage direction and the surface projection of the sentinel lymph nodes were marked on the skin for intraoperative guidance.

All patients underwent breast and axillary surgery within 3–4 weeks after completion of NAC. SLNB followed by ALND was performed in all patients. Breast surgery consisted of either breast-conserving surgery or mastectomy.

During the operation, 1 mL of 1% methylene blue solution was injected into the periareolar region on the affected side, followed by local massage for 15 min to facilitate lymphatic drainage of the dye. During surgery, CEUS-localized nodes, methylene blue-identified nodes, and any additional suspicious nodes identified by the surgeon were excised whenever technically feasible and submitted for pathological examination. The surgeon identified blue-stained lymph nodes in the axilla, assessed their concordance with the preoperative CEUS findings, and excised the sentinel lymph nodes. When the preoperative CEUS skin marking and intraoperative methylene blue mapping were discordant, both the CEUS-localized area/node and blue-stained lymph nodes were explored.

Patients were carefully monitored during and immediately after CEUS examination and intraoperative methylene blue mapping for procedure-related adverse events, including allergic reactions. Delayed adverse events after discharge were not systematically captured during follow-up.

### 2.6. Histopathological Evaluation

All resected SLNs were submitted separately for pathological examination and serially sliced at 2 mm intervals along their longest axis according to a standardized institutional protocol. Hematoxylin and eosin (H&E) staining was routinely performed on all tissue sections. Immunohistochemical staining was additionally performed by two experienced pathologists when H&E findings were equivocal or suspicious for metastatic involvement, including suspected micrometastases or isolated tumor cells. Nodal metastatic deposits were classified according to their maximum dimension as macrometastases (>2 mm), micrometastases (>0.2 mm and ≤2 mm), or isolated tumor cells (≤0.2 mm). The same pathological processing and diagnostic criteria were applied at both participating centers. The total number of lymph nodes, the number of positive lymph nodes, and the metastatic status in both sentinel lymph node and ALND specimens were recorded. pCR was defined as the absence of residual invasive disease in both the breast and axillary tissues (ypT0/is ypN0). Breast cancer molecular subtypes were determined according to the ASCO/CAP guidelines. Estrogen receptor (ER) and progesterone receptor (PR) positivity were defined as nuclear staining in ≥1% of tumor cells. HER2 status was determined based on immunohistochemistry and in situ hybridization results according to the ASCO/CAP recommendations. On this basis, patients were classified into four molecular subtypes: HR+/HER2−, HR−/HER2−, HR+/HER2+, and HR−/HER2+.

### 2.7. Statistical Analysis

Statistical analyses were performed using R software (version 4.3.2). Continuous variables are presented as the median (range) or median with interquartile range (IQR), as appropriate, and categorical variables are expressed as numbers and percentages. For the three mapping methods, sentinel lymph node detection rate (DR), false-negative rate (FNR), positive predictive value (PPV), and negative predictive value (NPV) were calculated separately. For the diagnostic performance of CEUS in predicting sentinel lymph node metastasis, sensitivity, specificity, PPV, NPV, accuracy, and overall concordance rate were calculated, using ALND pathology as the reference standard. Pairwise differences in SLN detection rates were assessed using exact McNemar tests based on discordant pairs. *p* values were adjusted for the three pairwise comparisons using the Holm method. Differences in FNR between subgroups were assessed using Fisher’s exact test. To evaluate the uncertainty of the FNR estimate, the one-sided 95% upper confidence limit (UCL) was calculated using the Wilson score method. All statistical tests were two-sided, except for the one-sided UCL estimation, and a *p* value < 0.05 was considered statistically significant.

## 3. Results

### 3.1. Patient Enrollment and Baseline Characteristics

During the study period, 471 patients were screened for eligibility at the two participating centers. After the exclusion of 202 patients, 269 patients were included in the analysis: 208 with initially cN1 disease, 45 with cN2 disease, and 16 with cN3 disease. All included patients underwent preoperative CEUS, intraoperative methylene blue mapping, SLNB, and ALND and were included in the diagnostic-performance analyses.

Overall, the median age of the study population was 50 years (range, 22–74 years), and premenopausal women accounted for more than half of the cohort. Baseline breast tumor burden was predominantly cT2, and after NAC, 131 patients (48.70%) achieved breast pCR and 178 (66.17%) achieved axillary pCR.

When stratified by initial nodal stage, the cN1 cohort remained the predominant subgroup. In these patients, the median age was 50 years (range, 22–74 years), 60.58% were premenopausal, and 73.56% presented with cT2 disease. Most cN1 patients were classified as clinical stage II before NAC (76.92%). After NAC treatment, breast and axillary pCR were observed in 97 (46.63%) and 142 patients (68.27%), respectively. By comparison, axillary pCR was lower in the cN2 cohort (48.89%), whereas the cN3 subgroup, despite the limited sample size, showed a relatively high axillary pCR rate in 14 of 16 patients (87.50%). In terms of molecular subtype, HR+/HER2− was the most common subtype in the cN1 cohort (36.54%), followed by HR−/HER2+ (32.69%). Detailed baseline clinicopathological characteristics of the study population are summarized in Table 1.

### 3.2. SLN Detection by CEUS, Methylene Blue, and the Combined Approach

The SLN detection outcomes are presented in Table 2. The combined CEUS plus methylene blue approach achieved the highest detection rate, with successful identification of at least one SLN in 206 of 208 patients (99.04%, 95% CI, 96.56–99.74), followed closely by CEUS alone in 204 of 208 patients (98.08%, 95% CI, 95.16–99.25). In contrast, methylene blue alone showed a substantially lower detection rate of 71.63% (149/208; 95% CI, 65.16–77.33). Pairwise exact McNemar tests showed higher detection rates for both CEUS alone and the combined approach than for methylene blue alone (both Holm-adjusted *p* < 0.001). The combined approach detected SLNs in two additional patients compared with CEUS alone, but the difference was not statistically significant (Holm-adjusted *p* = 0.500).

The median number of retrieved SLNs was 3 (IQR, 1–4) for both the combined approach and CEUS alone, compared with 2 (IQR, 1–4) for methylene blue alone. Under the combined approach, no SLN was detected in 2 patients, while 55, 47, 45, and 32 patients had 1, 2, 3, and 4 SLNs retrieved, respectively; 27 patients had 5 or more SLNs retrieved. Overall, these findings indicate that CEUS provided a markedly higher detection yield than methylene blue alone, and the combined approach had the highest observed SLN detection rate, although its detection rate did not differ significantly from that of CEUS alone.

### 3.3. Diagnostic Performance of CEUS, Methylene Blue, and the Combined Approach

With respect to false-negative results, the combined approach and CEUS alone yielded the same false-negative rate of 9.38% (95% CI, 4.37–18.98), which was numerically lower than that of methylene blue alone (12.50%, 95% CI, 5.46–26.11). However, the confidence intervals were relatively wide, indicating uncertainty in the precision of these estimates. The NPV remained high across all three methods, reaching 95.95% (95% CI, 91.44–98.13) for the combined approach, 95.89% (95% CI, 91.32–98.10) for CEUS alone, and 95.61% (95% CI, 90.14–98.11) for methylene blue alone. In addition, the PPV was comparable for the combined approach and CEUS alone (both 82.86%; 95% CI, 72.38–89.91) and slightly lower for methylene blue alone (81.40%; 95% CI, 67.38–90.26). These findings suggest that CEUS-based mapping, either alone or in combination with methylene blue, maintained a relatively low false-negative rate while preserving high predictive performance. Overall, the main advantage of combining CEUS with methylene blue was reflected primarily in improved SLN detection rather than a clearly lower FNR compared with CEUS alone. The diagnostic performance of the three mapping strategies is summarized in Table 3.

### 3.4. Subgroup Analysis of the Combined CEUS Plus Methylene Blue Approach

In patients with initial cN1 disease, the combined CEUS plus methylene blue approach maintained a high SLN detection rate across all subgroups, ranging from 97.92% to 100.00%. Detection performance was generally comparable across subgroups stratified by age, hormone receptor status, HER2 status, and clinical stage before NAC, indicating consistently high observed detection rates across the clinicopathological subgroups.

In contrast, the false-negative rate varied across subgroups. When stratified by age, patients aged <50 years had a higher FNR than those aged ≥50 years (22.22% vs. 0), and this difference was statistically significant (*p* = 0.003); correspondingly, the NPV reached 100.00% in patients aged ≥50 years. By comparison, although numerical differences in FNR were observed according to the number of retrieved SLNs, HR status, HER2 status, and clinical stage before NAC, none of these comparisons reached statistical significance (Table 4). Overall, the combined approach achieved consistently high detection rates in initially cN1 patients, while age appeared to be a potential factor associated with its false-negative performance. Given the small number of false-negative events and the exploratory nature of the subgroup comparisons, the observed association with age should be considered hypothesis-generating.

### 3.5. Concordance Between CEUS Localization and Methylene Blue Mapping

**Figure 2:**
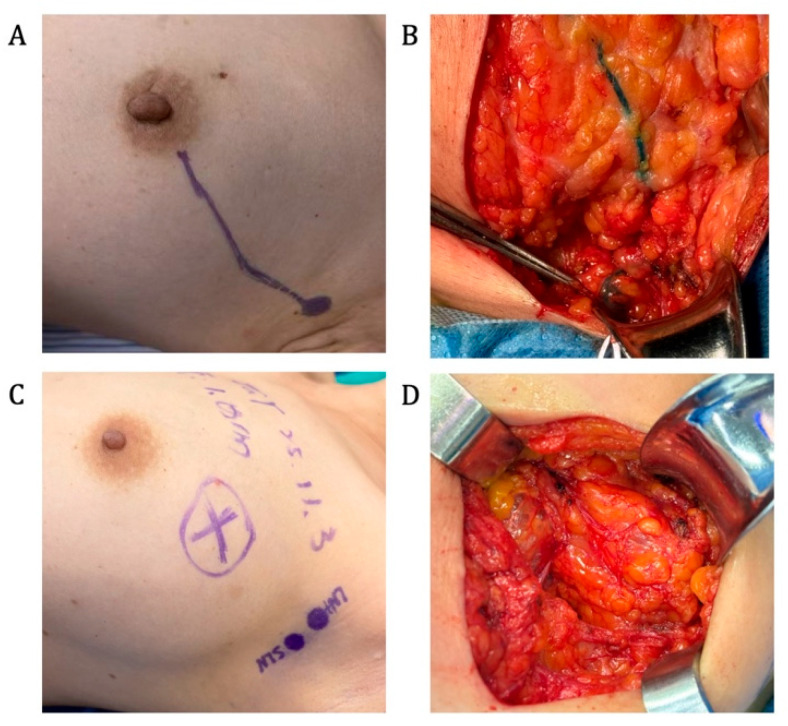
Representative cases of concordance and discordance between preoperative CEUS localization and intraoperative methylene blue mapping. (**A**,**B**) Representative photographs demonstrating concordance between the skin surface projection marked according to preoperative CEUS and the position of methylene blue–identified sentinel lymph nodes during surgery. (**C**,**D**) Representative photographs demonstrating discordance between the preoperative CEUS surface marking and intraoperative methylene blue localization. CEUS, contrast-enhanced ultrasound.

Concordance between CEUS localization and intraoperative methylene blue mapping could be assessed only when both methods successfully identified SLNs. Among the 149 patients with methylene blue-detected SLNs, 147 also had SLNs identified by CEUS and were therefore evaluable for concordance analysis. The remaining patients were not included in the concordance analysis because SLNs were not detected by one of the two methods, rather than because of missing procedural documentation. Preoperative CEUS localization was concordant with intraoperative methylene blue mapping in 134 of 147 cases, corresponding to a concordance rate of 91.16%. Discordance was observed in 13 patients (Figure 2). These findings indicate a high level of correspondence between CEUS-based preoperative localization and intraoperative methylene blue guidance at the patient level (Table 5).

### 3.6. Diagnostic Value of CEUS for Predicting SLN Metastasis

Among the 204 evaluable patients who successfully underwent preoperative CEUS, CEUS-based assessment of SLN metastatic status was concordant with ALND pathological findings in 164 cases, corresponding to an overall concordance rate of 80.39% (95% CI, 74.38–85.28), whereas discordance was observed in 40 cases. In terms of diagnostic performance, CEUS yielded a sensitivity of 65.52% (95% CI, 52.68–76.42), a specificity of 86.30% (95% CI, 79.80–90.93), a PPV of 65.52% (95% CI, 52.68–76.42), a NPV of 86.30% (95% CI, 79.80–90.93), and an overall accuracy of 80.39% (95% CI, 74.38–85.28) (Table 6). These findings suggest that CEUS showed relatively good agreement with pathological axillary status and may provide supplementary preoperative information on nodal metastatic status.

### 3.7. Exploratory Diagnostic Analysis in Initial cN2 and cN3 Patients

Because the primary analysis focused on initially cN1 patients, patients with initial cN2–3 disease were analyzed only exploratorily to assess the potential performance boundaries of CEUS combined with methylene blue in patients with higher nodal burden (Table 7). In the cN2 subgroup, the combined CEUS plus methylene blue approach achieved a detection rate of 91.11% (41/45; 95% CI, 79.27–96.49), which was higher than that of CEUS alone (84.44%; 38/45; 95% CI, 71.22–92.25) and methylene blue alone (66.67%; 30/45; 95% CI, 52.07–78.64). The corresponding false-negative rates were 33.33% (95% CI, 17.97–53.29), 31.82% (95% CI, 16.36–52.68), and 29.41% (95% CI, 13.28–53.13), respectively.

In the cN3 subgroup, the combined approach, CEUS alone, and methylene blue alone yielded detection rates of 100.00% (16/16; 95% CI, 80.64–100.00), 93.75% (15/16; 95% CI, 71.67–98.89), and 75.00% (12/16; 95% CI, 50.50–89.82), respectively, whereas the false-negative rate was 0 for all three methods; however, the confidence intervals were wide, reflecting substantial statistical uncertainty due to the small sample size. When patients with cN1–3 disease were analyzed together, the overall detection rate of the combined approach was 97.77% (263/269; 95% CI, 95.29–98.98), compared with 95.54% for CEUS alone (257/269; 95% CI, 92.39–97.42) and 71.00% for methylene blue alone (191/269; 95% CI, 65.31–76.06). The corresponding false-negative rates were 15.56% (95% CI, 9.46–24.47), 14.77% (95% CI, 8.78–23.60), and 16.95% (95% CI, 9.44–28.52), respectively.

Overall, although the combined strategy had a higher observed detection rate in patients with initial cN2–3 disease, these subgroup results should be interpreted cautiously because of the relatively small sample sizes and wide confidence intervals. The false-negative performance appeared less stable than that observed in the primary cN1 cohort, particularly in cN2 patients.

### 3.8. Adverse Events

No allergic reactions or other immediate or perioperative adverse events related to CEUS or methylene blue mapping were recorded. Delayed adverse events after discharge were not systematically assessed.

## 4. Discussion

In this prospective two-center study, CEUS combined with methylene blue achieved an SLN detection rate of 99.04% (95% CI, 96.56–99.74) in patients with initially cN1 breast cancer after NAC. The detection rates of both CEUS alone and the combined approach were significantly higher than that of methylene blue alone, whereas the difference between CEUS alone and the combined approach was not statistically significant. In addition, the combined approach yielded an overall FNR of 9.38% (95% CI, 4.37–18.98), showing a promising but statistically imprecise false-negative profile due to the relatively wide confidence interval and the underpowered final sample size. Preoperative CEUS localization was highly concordant with intraoperative methylene blue mapping and provided information on nodal metastatic status. The exploratory cN2–3 analyses showed greater uncertainty and less consistent false-negative performance. Taken together, these findings support CEUS combined with methylene blue as a feasible non-radioactive strategy for axillary restaging after NAC, particularly in patients with initial cN1 disease.

The improved detection performance of the combined approach is likely attributable to the complementary roles of CEUS and methylene blue in both timing and information acquisition. CEUS allows dynamic preoperative visualization of lymphatic drainage pathways from the areolar region to the axilla and enables surface localization of the first enhanced lymph node, thereby providing a more precise anatomical reference for intraoperative SLN exploration. In contrast, methylene blue directly delineates stained lymphatic channels and blue-stained lymph nodes during surgery, facilitating visual identification and excision under an open surgical field. Therefore, these complementary roles may explain the higher observed detection rate of the combined approach. However, its reproducibility and generalizability across centers with different levels of CEUS expertise remain to be established.

Compared with the detection rate, the FNR is more directly relevant to determining whether SLNB can reliably support omission of ALND after NAC, because it reflects the probability that residual axillary disease is missed despite a negative SLN finding. Pivotal studies prespecified an FNR of 10% as a benchmark, reflecting the expected performance of SLNB in clinically node-negative patients [11,15]. In initially node-positive patients treated with NAC, reported FNRs range from approximately 4% to 14%, depending on the mapping technique, pathological assessment, clipped-node retrieval, and use of targeted axillary dissection [11,12,13,14,15,16,17,18,19,20]. In our cN1 cohort, the FNR point estimate was 9.38%; however, the two-sided 95% CI ranged from 4.37% to 18.98% and its upper bound clearly exceeded the 10% benchmark. Moreover, the one-sided 95% upper confidence limit was 17.12%. Together with the underpowered final sample size, this statistical uncertainty prevents us from concluding that the true FNR is ≤10% or that CEUS combined with methylene blue is equivalent to radioisotope-based dual-tracer mapping or targeted axillary dissection. Thus, our findings support the feasibility and diagnostic promise of CEUS combined with methylene blue, but larger comparative studies are required before this strategy can be used to guide routine omission of ALND.

In this prospective study, the detection rate improved from 71.63% to 99.04%, which means that the combined approach may increase the number of patients who may technically be considered for omission of ALND. Based on our data, the combined approach would increase the number of patients entering the technical eligibility range by approximately 27 per 100 patients for potential ALND omission while still carrying a measurable false-negative risk (9.38%). However, increasing evidence suggests that, in carefully selected patients with initially cN1 disease who convert to ycN0 after NAC, omission of ALND after negative SLNB may still be associated with very low axillary failure under contemporary multimodality treatment. In the Memorial Sloan Kettering Cancer Center (MSKCC) cohort, the axillary recurrence rate was only 0.4% (1/234) at a median follow-up of 40 months, and no nodal recurrences were observed among patients who received adjuvant radiotherapy [5]. A subsequent multicenter cohort study further reported a 5-year axillary recurrence rate of approximately 1.0% after omission of ALND in this setting [6]. Moreover, recent meta-analyses have shown favorable 5-year survival and low axillary recurrence rates among initially node-positive patients who achieve nodal pCR after NAC and undergo SLNB without ALND [29,30]. Taken together, these findings suggest that a certain probability of technical false-negative events may not necessarily translate into clinically unacceptable axillary failure. However, because long-term axillary recurrence, disease-free survival, and overall survival were not assessed in the present cohort, our study should be regarded as evidence of technical feasibility and promising diagnostic performance, rather than direct evidence supporting ALND omission.

In an exploratory subgroup analysis, age appeared to be associated with false-negative performance. Patients younger than 50 years had a significantly higher FNR than those aged 50 years or older (22.22% vs. 0, *p* = 0.003), despite similarly high detection rates across age groups. Although this observation should be interpreted cautiously because of the limited number of false-negative events, it raises the possibility that patient age may influence the reliability of post-NAC SLNB. One potential explanation is that younger patients are more likely to harbor biologically aggressive disease, which may be associated with a more heterogeneous pattern of residual nodal involvement after treatment. In addition, tumor biology and treatment-induced alterations in lymphatic drainage or residual disease distribution may differ across age groups and thereby affect the representativeness of the retrieved sentinel nodes [31]. Further studies with larger sample sizes are warranted to determine whether age is a true modifier of false-negative performance.

We further included patients with initial cN2–3 disease in an exploratory analysis to examine the potential boundaries of this strategy in the setting of a higher initial axillary tumor burden. In this subgroup, the combined CEUS plus methylene blue approach still achieved a higher detection rate than methylene blue alone, suggesting that the complementary value of preoperative CEUS and intraoperative dye mapping may extend beyond cN1 disease at the technical level. However, the small sample sizes and wide confidence intervals preclude firm conclusions regarding performance in cN2–3 disease. These findings suggest that as the initial nodal burden increases, post-NAC lymphatic drainage patterns and the distribution of residual nodal disease may become more complex, thereby limiting the representativeness of sentinel lymph nodes [32]. Rather than supporting direct extrapolation of this strategy to higher-burden nodal disease, these exploratory data reinforce initial cN1 disease as the most appropriate target population for further evaluation.

Beyond its role in SLN mapping, CEUS also demonstrated adjunctive value in predicting nodal metastatic status. In the present study, CEUS achieved an overall accuracy of 80.39%, with a specificity of 86.30% and an NPV of 86.30%, indicating moderate supplementary value for identifying patients without residual nodal metastasis. In this sense, CEUS may contribute not only anatomical guidance for SLN localization but also supplementary functional information for preoperative nodal assessment. However, the sensitivity was only 65.52%, indicating that a negative or nonsuspicious CEUS assessment cannot reliably exclude residual nodal metastasis. This limitation may be particularly relevant after NAC, as treatment-related fibrosis, necrosis, and changes in vascularity or perfusion can alter enhancement behavior and contribute to discordance between imaging findings and true pathological status. Therefore, CEUS should currently be regarded as an adjunct rather than a substitute for pathological assessment.

This study has several limitations. First, although it was prospective and conducted at two centers, both centers are affiliated with the same university system and used a standardized CEUS workflow performed by experienced breast-imaging specialists. The generalizability of the findings to community hospitals, lower-volume institutions, and centers with less experience in CEUS-guided SLN mapping therefore remains uncertain. Second, enrollment was terminated early following ethical review, leaving the final cohort underpowered for precise estimation of the primary FNR endpoint. Third, the study did not include a radioisotope-based dual-tracer control group. Consequently, the findings cannot establish equivalence or non-inferiority relative to standard radioisotope-guided mapping. Fourth, long-term oncologic outcomes, including axillary recurrence, disease-free survival, and overall survival, were not assessed; therefore, the study cannot determine whether this strategy can safely guide the omission of ALND after NAC. Finally, CEUS acquisition and interpretation remain operator-dependent, which may limit reproducibility across centers.

Despite these limitations, the present study has practical clinical relevance. In settings where radioisotope-guided mapping is unavailable, CEUS combined with methylene blue may offer a more accessible non-radioactive approach to SLN mapping. This combined approach achieved a high detection performance, a promising false-negative profile and may provide additional information for preoperative axillary restaging. The method does not require nuclear medicine facilities, a radioisotope supply, or radiation-related regulatory procedures. Methylene blue is inexpensive and widely available, and CEUS can be performed using ultrasound systems equipped with contrast-specific imaging capabilities. Therefore, this approach may have potential cost and accessibility advantages in resource-limited settings. These findings should be viewed as supportive evidence for further investigation rather than validation for widespread clinical implementation or direct guidance of axillary surgical de-escalation.

## 5. Conclusions

In patients with initially cN1 breast cancer after neoadjuvant chemotherapy, CEUS combined with methylene blue achieved high SLN detection and showed a promising false-negative profile without radioisotope guidance. CEUS also demonstrated high concordance with intraoperative methylene blue mapping and provided supplementary value for predicting SLN metastasis. These findings should be interpreted as preliminary evidence of feasibility and diagnostic performance. Larger comparative studies are needed before widespread clinical implementation or use in guiding axillary surgical de-escalation.

## Figures and Tables

**Table 1:** Baseline clinicopathological characteristics of the study population.

Characteristics	cN1, *n* (%)	cN2, *n* (%)	cN3, *n* (%)	Total, *n* (%)
*n*	208	45	16	269
Age, median (range), years	50 (22–74)	51 (24–68)	50 (25–66)	50 (22–74)
Age < 50	100 (48.08)	15 (33.33)	7 (43.75)	122 (45.35)
Age ≥ 50	108 (51.92)	30 (66.67)	9 (56.25)	147 (54.65)
BMI (kg/m^2^)				
<25	141 (67.79)	32 (71.11)	12 (75.00)	185 (68.77)
≥25	67 (32.21)	13 (28.89)	4 (25.00)	84 (31.23)
Menstrual status				
Premenopausal	126 (60.58)	18 (40.00)	8 (50.00)	152 (56.51)
Menopausal	82 (39.42)	27 (60.00)	8 (50.00)	117 (43.49)
Clinical T stage				
cT0	0	1 (2.22)	0	1 (0.37)
cT1	10 (4.81)	4 (8.89)	0	14 (5.20)
cT2	153 (73.56)	30 (66.67)	10 (62.50)	193 (71.75)
cT3	45 (21.63)	10 (22.22)	5 (31.25)	60 (22.30)
cT4	0	0	1 (6.25)	1 (0.37)
Clinical TNM stage prior NAC				
II	160 (76.92)	0	0	160 (59.48)
III	48 (23.08)	45 (100)	16 (100)	109 (40.52)
Pathological breast status after NAC				
ypT0	97 (46.63)	25 (55.56)	9 (56.25)	131 (48.70)
ypT1	74 (35.58)	13 (28.89)	5 (31.25)	92 (34.20)
ypT2	30 (14.42)	7 (15.56)	2 (12.50)	39 (14.50)
ypT3	2 (0.96)	0	0	2 (0.74)
ypT4	4 (1.92)	0	0	4 (1.49)
Pathological axillary status after NAC				
ypN0	142 (68.27)	22 (48.89)	14 (87.50)	178 (66.17)
ypN1	42 (20.19)	13 (28.89)	1 (6.25)	56 (20.82)
ypN2	13 (6.25)	7 (15.56)	1 (6.25)	21 (7.81)
ypN3	10 (4.81)	3 (6.67)	0	13 (4.83)
Molecular subtypes				
HR+/HER2−	76 (36.54)	18 (40.00)	5 (31.25)	99 (36.80)
HR−/HER2−	30 (14.42)	8 (17.78)	5 (31.25)	43 (15.99)
HR+/HER2+	34 (16.35)	9 (20.00)	5 (31.25)	48 (17.84)
HR−/HER2+	68 (32.69)	10 (22.22)	1 (6.25)	79 (29.37)
Breast surgery				
Lumpectomy	45 (21.63)	12 (26.67)	1 (6.25)	58 (21.56)
Mastectomy	163 (78.37)	33 (73.33)	15 (93.75)	211 (78.44)
Breast pCR				
Yes	97 (46.63)	25 (55.56)	9 (56.25)	131 (48.70)
No	111 (53.37)	20 (44.44)	7 (43.75)	138 (51.30)
Axillary pCR				
Yes	142 (68.27)	22 (48.89)	14 (87.50)	178 (66.17)
No	66 (31.73)	23 (51.11)	2 (12.50)	91 (33.83)
Axillary pCR by subtypes				
HR+/HER2−	32 (22.54)	3 (6.67)	3 (18.75)	38 (14.13)
HR−/HER2−	24 (16.90)	3 (6.67)	5 (31.25)	32 (11.90)
HR+/HER2+	24 (16.90)	6 (13.33)	5 (31.25)	35 (13.01)
HR−/HER2+	62 (43.66)	10 (22.22)	1 (6.25)	73 (27.14)

Abbreviations: BMI, body mass index; cT, clinical tumor stage; TNM, tumor-node-metastasis; HER2, human epidermal growth factor receptor 2; HR, hormone receptor; NAC, neoadjuvant chemotherapy; pCR, pathological complete response; ypT, pathological tumor stage after NAC; ypN, pathological lymph node status after NAC.

**Table 2:** Detection rate and number of retrieved sentinel lymph nodes by CEUS, methylene blue, and the combined approach.

Characteristics	Value
Detection rate, *n*/N (%, 95% CI)	
CEUS + methylene blue	206/208 (99.04, 95% CI, 96.56–99.74)
CEUS	204/208 (98.08, 95% CI, 95.16–99.25)
Methylene blue	149/208 (71.63, 95% CI, 65.16–77.33)
Number of SLNs retrieved, median (IQR)	
CEUS + methylene blue	3 (1–4)
CEUS	3 (1–4)
Methylene blue	2 (1–4)
CEUS + methylene blue number of SLNs retrieved, *n*	
0	2
1	55
2	47
3	45
4	32
≥5	27

Abbreviations: CEUS, contrast-enhanced ultrasound; SLNs, sentinel lymph nodes; IQR, interquartile range.

**Table 3:** Diagnostic performance of CEUS, methylene blue, and the combined approach in cN1 patients.

Mapping Method	FNR (%)	95% CI	NPV (%)	95% CI	PPV (%)	95% CI
CEUS + methylene blue	9.38	4.37–18.98	95.95	91.44–98.13	82.86	72.38–89.91
CEUS	9.38	4.37–18.98	95.89	91.32–98.10	82.86	72.38–89.91
Methylene blue	12.50	5.46–26.11	95.61	90.14–98.11	81.40	67.38–90.26

Abbreviations: CEUS, contrast-enhanced ultrasound; FNR, false-negative rate; NPV, negative predictive value; PPV, positive predictive value.

**Table 4:** Subgroup analysis of the combined CEUS plus methylene blue approach in cN1 patients.

	Patients, *n*	DR (%)	FNR (%)	95% UCL for FNR (%)	*p* Value	NPV (%)
Age (years)						
<50	100	99.00	22.22	37.55	0.003	94.81
≥50	108	99.07	0	6.65		100.00
Number of SLNs removed						
1	55	98.11	11.11	28.64	1	94.44
2	47	97.92	0	25.28		100.00
≥3	104	100.00	10.53	21.39		94.29
HR status						
HR+	110	98.18	9.62	18.47	1	91.80
HR−	98	100	8.33	30.12		98.85
HER2 status						
HER2+	102	100	6.25	23.75	1	98.85
HER2−	106	98.11	10.42	19.90		91.80
Clinical TNM stage before NAC						
II	160	99.38	10.64	20.29	0.66	95.76
III	48	97.92	5.26	20.50		96.67

Abbreviations: CEUS, contrast-enhanced ultrasound; DR, detection rate; FNR, false-negative rate; UCL, upper confidence limit; NPV, negative predictive value; SLNs, sentinel lymph nodes; HR, hormone receptor; HER2, human epidermal growth factor receptor 2; TNM, tumor-node-metastasis; NAC, neoadjuvant chemotherapy.

**Table 5:** Concordance between CEUS localization and intraoperative methylene blue mapping in cN1 patients.

Variable	Value
Total evaluable patients	147
Concordant cases	134
Discordant cases	13
Concordance rate (%)	91.16

**Table 6:** Diagnostic performance of CEUS for predicting sentinel lymph node metastasis in cN1 patients.

Variable	Value
Total evaluable patients	204
Concordant cases	164
Discordant cases	40
Concordance rate (%)	80.39 (95% CI, 74.38–85.28)
Sensitivity (%)	65.52 (95% CI, 52.68–76.42)
Specificity (%)	86.30 (95% CI, 79.80–90.93)
PPV (%)	65.52 (95% CI, 52.68–76.42)
NPV (%)	86.30 (95% CI, 79.80–90.93)
Accuracy (%)	80.39 (95% CI, 74.38–85.28)

Abbreviations: NPV, negative predictive value; PPV, positive predictive value.

**Table 7:** Diagnostic performance for three approaches in cN2 and cN3 patients.

Mapping Method	Patients, n/N	DR (%, 95% CI)	FNR (%, 95% CI)	NPV (%, 95% CI)	PPV (%, 95% CI)
cN2					
CEUS + methylene blue	41/45	91.11 (79.27–96.49)	33.33 (17.97–53.29)	68.00 (48.41–82.80)	72.73 (51.85–86.85)
CEUS	38/45	84.44 (71.22–92.25)	31.82 (16.36–52.68)	69.57 (49.13–84.40)	71.43 (50.04–86.19)
Methylene blue	30/45	66.67 (52.07–78.64)	29.41 (13.28–53.13)	72.22 (49.13–87.50)	70.59 (46.87–86.72)
cN3					
CEUS + methylene blue	16/16	100.00 (80.64–100.00)	0 (0.00–65.76)	100 (78.47–100.00)	66.67 (20.77–93.85)
CEUS	15/16	93.75 (71.67–98.89)	0 (0.00–65.76)	100 (72.25–100.00)	66.67 (20.77–93.85)
Methylene blue	12/16	75.00 (50.50–89.82)	0 (0.00–65.76)	100 (77.19–100.00)	66.67 (20.77–93.85)
cN1 + cN2 + cN3					
CEUS + methylene blue	263/269	97.77 (95.29–98.98)	15.56 (9.46–24.47)	92.51 (87.71–95.53)	80.00 (70.80–86.87)
CEUS	257/269	95.54 (92.39–97.42)	14.77 (8.78–23.60)	92.90 (87.92–95.98)	79.79 (70.43–86.73)
Methylene blue	191/269	71.00 (65.31–76.06)	16.95 (9.44–28.52)	92.96 (87.31–96.17)	77.78 (65.80–86.52)

Abbreviations: CEUS, contrast-enhanced ultrasound; DR, detection rate; FNR, false-negative rate; NPV, negative predictive value; PPV, positive predictive value; cN, clinical lymph node status before neoadjuvant chemotherapy.

## Data Availability

The data that support the findings of this study are available from the corresponding authors upon reasonable request.

## Abbreviations

The following abbreviations are used in this manuscript:

NAC	Neoadjuvant chemotherapy
SLNB	Sentinel lymph node biopsy
ALND	Axillary lymph node dissection
SLN	Sentinel lymph node
CEUS	Contrast-enhanced ultrasound
pCR	Pathological complete response
FNR	False-negative rate
DR	Detection rate
PPV	Positive predictive value
NPV	Negative predictive value
HE	Hematoxylin–eosin
ER	Estrogen receptor
PR	Progesterone receptor
HER2	Human epidermal growth factor receptor 2
TNM	Tumor-node-metastasis
IQR	Interquartile range
UCL	Upper confidence limit
BMI	Body mass index
